# Novel Therapeutic Opportunities in Neoadjuvant Setting in Urothelial Cancers: A New Horizon Opened by Molecular Classification and Immune Checkpoint Inhibitors

**DOI:** 10.3390/ijms23031133

**Published:** 2022-01-20

**Authors:** Maria Lucia Iacovino, Chiara Carmen Miceli, Marco De Felice, Biagio Barone, Luca Pompella, Francesco Chiancone, Erika Di Zazzo, Giuseppe Tirino, Carminia Maria Della Corte, Ciro Imbimbo, Ferdinando De Vita, Felice Crocetto

**Affiliations:** 1 Department of Precision Medicine, Medical Oncology, University of Campania Luigi Vanvitelli, Via Sergio Pansini 5, 80131 Naples, Italy; marialucia.iacovino@unicampania.it (M.L.I.); chiaracarmen.miceli@unicampania.it (C.C.M.); marco.defelice@unicampania.it (M.D.F.); luca.pompella@unicampania.it (L.P.); giuseppe.tirino@unicampania.it (G.T.); carminiamaria.dellacorte@unicampania.it (C.M.D.C.); ferdinando.devita@unicampania.it (F.D.V.); 2 Department of Neurosciences, Reproductive Sciences and Odontostomatology, University of Naples “Federico II”, 80131 Naples, Italy; biagio.barone@unina.it (B.B.); ciro.imbimbo@unina.it (C.I.); 3 Department of Urology, AORN “A.Cardarelli”, 80131 Naples, Italy; francescok86@gmail.com; 4 Department of Medicine and Health Sciences “V. Tiberio”, University of Molise, UOC Laboratorio Analisi P.O. “A. Cardarelli”, 86100 Campobasso, Italy; erika.dizazzo@unimol.it

**Keywords:** bladder cancer, urothelial cancers, neoadjuvant chemotherapy, Immune Checkpoint Inhibitors, molecular classification of muscle-invasive bladder cancer, muscle-invasive bladder cancer

## Abstract

Muscle invasive bladder cancer (MIBC) is a widespread malignancy with a worse prognosis often related to a late diagnosis. For early-stage MIBC pts, a multidisciplinary approach is mandatory to evaluate the timing of neoadjuvant chemotherapy (NAC) and surgery. The current standard therapy is platinum-based NAC (MVAC-methotrexate, vinblastine, doxorubicin, and cisplatin or Platinum–Gemcitabine regimens) followed by radical cystectomy (RC) with lymphadenectomy. However, preliminary data from Vesper trial highlighted that dose-dense NAC MVAC is endowed with a good pathological response but shows low tolerability. In the last few years, translational-based research approaches have identified several candidate biomarkers of NAC esponsiveness, such as ERCC2, ERBB2, or DNA damage response (DDR) gene alterations. Moreover, the recent consensus MIBC molecular classification identified six molecular subtypes, characterized by different sensitivity to chemo- or targeted or immunotherapy, that could open a novel procedure for patient selection and also for neoadjuvant therapies. The Italian PURE-01 phase II Trial extended data on efficacy and resistance to Immune Checkpoint Inhibitors (ICIs) in this setting. In this review, we summarize the most relevant literature data supporting NAC use in MIBC, focusing on novel therapeutic strategies such as immunotherapy, considering the better patient stratification and selection emerging from novel molecular classification.

## 1. Introduction

Urothelial bladder cancer (BC) accounts for an estimated 500,000 new cases and 200,000 deaths worldwide, with more than 25,500 new cases in Italy estimated in 2020 [1].

BC is classified in nonmuscle invasive (NMIBC) (75% of diagnoses) [2] and muscle-invasive bladder cancer (MIBC). MIBC is characterized by pelvic and/or iliac lymph node metastases in a great percentage of patients (pts) and sometimes by distant metastasis, such as bone lesions [3]. Therefore, especially in MIBC, accurate staging is mandatory to apply the appropriate therapeutic strategy [4].

Since the 1980s, when cisplatin-based chemotherapy (CT) was introduced for MIBC treatment, only few steps forward have been taken. Although international guidelines recommend neoadjuvant chemotherapy (NAC) with cisplatin-based schemes, less than 20% of MIBC pts receive this treatment in clinical practice [5,6]. Firstly, 50% of subjects are unfit for CT, and carboplatin is applied for a subset of them [7]. Secondly, clinical and pathological predictive markers to identify pts eligible for NAC therapy are not available yet [8]. To improve clinical MIBC outcome, CT has been proposed in association with loco-regional treatments such as surgery (radical cystectomy- RC) and/or radiotherapy (RT).

In recent years, translational-based research approaches have made great efforts in building up the concept of personalized medicine for oncology pts, suggesting new prognostic and predictive biomarkers; novel targets, and introducing new therapies, such as immunotherapy-based ones. DNA and RNA sequencing of BC specimens showed the complex genetic heterogeneity of BC and identified specific profiles based on the type and frequency of mutations, on gene copy numbers and on the methylation patterns that could be helpful to define patient prognosis and sensitivity to specific treatments [9]. Tumor biology studies could be useful to discover novel early-stage disease and tumor progression markers. Specifically, MIBC harbors a higher overall mutation rate and number of chromosomal aberrations than NMIBC, mainly involving the activation of prosurvival pathways such as p53, Rb, Pi3k-mTor, and RAS [10].

## 2. Molecular Classifications of MIBC: A Yet Uncomplete Picture

BC is a very complex disease, both from a clinical and biological point of view. In our opinion, molecular biology represents the key to reveal the possible Achilles’ heel in BC and to identify novel targets in neoadjuvant as well as advanced settings.

In 2014, the first comprehensive bulk multiomics characterization of MIBC was carried out on The Cancer Genome Atlas (TCGA) samples [9]. Whole-genome and RNA-sequencing of 131 chemo-naïve MIBC was performed to detect somatic mutation, copy number aberrations (CNA), and transcriptomic subtypes. The analysis revealed 32 significantly mutated genes [SMGs] (9 of them identified for the first time in MIBC) and the majority (93%) were involved in cell cycle regulation, chromatin structure modification (89%), and kinase signaling pathways (72%). The most relevant mutated genes were involved in the PI3K-AKT-mTOR pathway (42% of tumors—PIK3CA, 17%; TSC1/2, 9%; AKT3, 10%) and RTK/RAS pathways (FGFR3 activation, 17%; EGFR amplification, 9%; ERBB3 mutations, 6%; ERBB2 mutations or amplifications, 9%). It is interesting to note that almost 69% of the altered genes involved in kinase signaling pathways could be targetable. On the other side—and complementary to findings from whole-genome sequencing—RNA-sequencing identified at least 4 transcriptomic subtypes; among them, the attention was focused on Cluster 1 (Papillary-like), which is characterized by FGFR3 gene aberrations (mutations, amplifications, rearrangements), potentially targetable with FGFR3 inhibitors, and Cluster 3 (basal/squamouslike), similar to basal-like breast cancers.

Three years later, TCGA research network increased MIBC’s sample size to 412 pts [11] and provided the most accurate bulk molecular classification currently available of MIBC with a multiomics approach (Whole-genome sequencing, Copy number analysis, DNA methylation analysis, mRNA-sequencing, noncoding RNA-sequencing). The analysis showed 58 SMGs (34 novel vs. analysis performed in 2014) and identified five mutational signatures (the most relevant was related to APOBEC-a and APOBEC-b, accounting for 67% of tumors), with a different pts survival. The RNA-sequencing approach revealed five different subtypes. Integration of the results achieved with the other platform allowed the correlation among each subtype with possible therapeutic targets and survival information.

Of note, three subtypes called “luminal” (due to the expression of markers such as KRT20, GATA3, and FOXA1) are distinct:

- “Luminal-papillary” (35%): Characterized by papillary morphology, FGFR3 mutations/amplifications/fusions, activation of Sonic-Hedgehog pathways, low risk of progression, and a low NAC response rate. The relative high frequency of FGFR3 gene aberrations suggested that this subtype could be targetable with FGFR3 (or PAN-FGFR) inhibitors, such as Erdafitinib. This drug has already been tested in a second-line setting in the phase II BLC2001 Trial [12] and was approved by the FDA in April 2019 for treatment of advanced BC with FGFR3 (or FGFR2) genetic alterations. A confirmatory phase III trial is currently ongoing (NCT03390504) in a second/third-line setting. This trial aims to compare Erdafitinib with standard chemotherapy (Docetaxel, Vinflunine) or immunotherapy (pembrolizumab, second line) in pts harboring FGFR aberrations.
- “Luminal-infiltrated” (19%): Characterized by low tumor purity with high immune/stromal infiltration and high expression of PDL1 and CTLA4. It has been speculated that this subtype could respond to immune checkpoint inhibition therapy, and could show no or few responses to standard platinum-based CT.
- “Luminal-subtype” (6%): Characterized by high expression levels of uroplakines, KRT20, and SNX31. Targets for this subtype have not been discovered yet.

Other subtypes are as follows:

- “Basal/Squamous” subtype (35%): mostly observed in women than men. It shows squamous differentiation (basal keratin markers) with high PDL1 and CTLA4 immune/stromal populations. It has been speculated that this subtype could respond well to both platinum-based CT and immunotherapy.
- The “neuronal” subtype (5%), the last identified one (a “novelty” of this classification): It expresses both neuroendocrine and neuronal genes with a high cell-cycle signature. This MIBC type does not exhibit the typical morphology of neuroendocrine tumors and RNA-seq or specific immunohistochemistry staining could identify it. In this case, the best therapeutic option could be—similar to other high-grade neuroendocrine tumors—a combination of platinum and etoposide.

A consensus for the transcriptomic classification recapitulating all published data, similar to that performed for colorectal cancer [13], was published in 2020 in the European Urology Journal [14]. The classification identified 6 consensus MIBCs: Luminal-papillary (LumP), Luminal-non-Specified (LumNS), Luminal-Unstable (LumU), Stroma-rich, Basal/Squamous (Ba/Sq), and Neuroendocrine-like (NE-like). Although this classification shows partial overlapping with the TCGA transcriptional subtypes, it adds clinical and molecular data, and represents a starting point for the design of new generation molecular-oriented clinical trials.

## 3. Neoadjuvant Chemotherapy (NAC): The State of Art

### 3.1. Clinical Staging

Clinical management of BC pts is complex and not well-standardized: multiple options could be selected to reach the effective and appropriate treatments for each patient [15]. BC is usually detected after the clinical observation of hematuria. Cystoscopy, showing high sensitivity and specificity up to 95% and 100%, and high positive and negative predictive value, represents the gold standard for diagnosis and follow-up. However, flat and papillary lesions could be missed and nodal/extravesical involvement could not be assessed [16,17]. Considering that TNM staging persists as an independent prognostic factor with a 5-years specific survival rate ranging from 96% for carcinoma in situ, to 36% for loco-regional disease, and 10% for metastatic disease, diagnostic imaging is pivotal in clinical practice for appropriate staging and for the therapeutic algorithm [18,19].

The latest American College of Radiology Appropriateness Criteria (ACRAC) report stated that pretreatment staging of MIBC should include imaging of the urothelial upper tract with Magnetic Resonance Imaging (MRI) of the pelvis for local staging or Computerized Tomography (CT) of the abdomen and pelvis without and with contrast to assess synchronous lesions of the urothelium and abdominal organs, plus imaging of the chest for excluding lung metastases [20].

However, morphology assessment showed low sensitivity and specificity in detecting pathological lymph nodes due to the occurrence of false positive results, because the main criteria used to deem a lymph node as suspicious for metastatic disease is its size. Current recommendations suggest a major diameter cut-off > 8 mm for pelvic lymph nodes and >1 cm for retroperitoneal ones [21]. Functional evaluation with diffusion-weighted and dynamic MRI sequences improves our ability to perform nodal staging compared with conventional MRI and could optimize treatment response assessment, distinguishing early responders to NAC [22,23,24]. By the way, Nguyen et al. developed a model to characterize the BC microcirculatory changes and, by comparing MR images acquired at the beginning and at mid-NAC, observed significant differences between responders and nonresponders [25].

Assessing the importance of functional diagnostics, the latest ACRAC highlights that 18-Fluorodeoxyglucose (FDG) Positron Emission Tomography (PET)/CT could be appropriate for BC staging. This technique shows high sensitivity for preoperative lymph node staging and extravesical involvement, particularly for sub-centimetric nodes, where the traditional size criterion cannot be helpful [15]. In addition, several data suggest a potential role of 18-FDG PET/CT and PERCIST Criteria for restaging and response evaluation of MIBC. Progression-free survival (PFS) was significantly higher in pts with negative scans versus those with persistence of disease after NAC, and overall survival (OS) significantly reduced in the case of positive scan [26].

### 3.2. Neoadjuvant CT

Nowadays, the standard therapy for non-metastatic MIBC pts consists of platinum-based NAC, followed by RC and bilateral pelvic lymphadenectomy. RC alone, in fact, is associated with high recurrence rates and poor prognosis (5-years OS up to 50%) [27]. Chemotherapy administered in a neoadjuvant setting shows some advantages: the ability to deliver effective systemic therapy for micro-metastatic disease; improvement of direct drug delivery into the bladder and to surrounding lymphatic vessels and lymph nodes; and increases the probability of a better performance status of naïve pts for major surgical procedure [28]. In this setting, MIBC pts treated with NAC showed a downgrade of disease and an overall improved prognosis. A recent metanalysis demonstrated a 5% OS and a 9% disease-free survival (DFS) improvement at 5 years [29]. However, SWOG and EORTC studies reported a high-grade toxicity in more than one patient out of three, without a significant OS improvement in MIBC pts. Thus, the selection of pts with clinical T3 and T4 that could benefit from NAC avoiding toxicity is a challenge for the future [29]. MIBC could be further divided into low- and high-risk groups, including >cT3b disease, presence of hydroureteronephrosis, lymphovascular invasion, or more aggressive variant histologies such as squamous, sarcomatoid, or neuroendocrine features [30].

The NAC regimen for MIBC treatment has not been modified over the years. Firstly, a single-agent platinum-based chemotherapy was proposed that was immediately discarded for lack of benefit (hazard-ratio (HR) = 1.15, *p* = 0.264) [31]. Therefore, in four following perspective trials, only platinum-based multiagent regimens were tested. However, all studies showed low accrual rate and the results obtained were not statistical significant to demonstrate a benefit over surgery upfront [32,33,34,35,36]. Subsequently, the Advanced Bladder Cancer meta-analysis Collaboration group combined the results of these trials in a metanalysis and observed that platinum-based regimens significantly improved OS (combined HR_0.86, *p* = 0.003), with a risk of death decreased by 13%, with an absolute benefit equal to 5% and an absolute DFS improved by 9% at 5 years, independently of the type of local treatment, and in all subgroups of pts. This meta-analysis also assessed that the evidence of ypT0 stage after RC was a valid surrogate marker of oncological outcomes improvement [31].

MVAC (methotrexate, vinblastine, doxorubicin, and cisplatin) in a high-dose-intensity schedule (every fourteen days plus granulocyte colony stimulating factor), for three cycles in six weeks and minimizing the interval between diagnosis and surgery, represents the first multiagents regimen studied. Interestingly, about half of the pts achieved pathological complete response (pCR) or a downstaging to NMIBC, and 82% of pts with cN1 disease before cystectomy were pN0 after surgery [37,38]. However, as previously pointed out, one of the issues in the multidisciplinary management of MIBC is the correct preoperative staging of lymph nodes. Thus, these conclusions should be interpreted with caution for the risk of radiological overstaging.

Over the past years, Gemcitabine and cisplatin (GC) combination has been evaluated as a possible alternative to MVAC regimen. One prospective randomized phase III trial including locally advanced (T4b, N2, N3) or metastatic MIBC, comparing GC with MVAC, demonstrated that GC has noninferior oncological outcomes and a safer toxicity profile than MVAC [39].

Retrospective trials offered variable results. One large retrospective analysis of 212 pts showed similar results in terms of efficacy and safety [40]; another retrospective review of 319 pts affected by urothelial cancer who received neoadjuvant chemotherapy and cystectomy conducted by Zargar et al. [41] showed better results with a dose-dense (dd) MVAC regimen in terms of local control, pathological complete response (ypT0N0), compared with the GC regimen (28% vs. 15%, *p* = 0.005).

The French GETUG/AFU V05 VESPER [42] trial was the first prospective randomized phase III controlled trial comparing the efficacy of dd-MVAC and GC in perioperative setting (before or after RC). The primary endpoint was PFS at 3 years. Secondary endpoints were response rate (RR) according to RECIST 1.1 criteria (in neoadjuvant setting), OS and time to progression (TTP), and chemotherapy toxicity.

First results on pathological responses and chemotherapy toxicity were published early this year [43]. A higher local control rate (pCR, tumor downstaging, or organ confined) was observed in the dd-MVAC arm (*p* = 0.021). Hematological toxicities were equally reported in both arms, but severe anemia was most frequent in the dd-MVAC arm (*p* < 0.001). Other common adverse events were asthenia and gastrointestinal disorders (nausea/vomiting), more frequently observed in the dd-MVAC arm.

Recently, data on PFS were presented [42], showing a three-years PFS of 64% in the dd-MVAC arm vs. 56% of GC (HR = 0.77; 95% CI 0.57–1.02; *p* = 0.066) considering the entirety of the perioperative setting. Further, considering the neoadjuvant group separately, three-years PFS was significantly higher for the dd-MVAC arm. Additionally, TTP was improved, and this met statistical significance (3-years rate: 69% vs. 58%, HR = 0.68 (95% CI 0.50–0.93), *p* = 0.014). dd-MVAC arm also improved OS in the neoadjuvant group (HR = 0.66; 95% CI 0.47–0.92).

Some relevant issues need to be discussed when considering neoadjuvant CT for MIBC.

Table 1 summarizes the main trials evaluating neoadjuvant chemotherapy for BC.

### 3.3. How Many Pts Are Fit for Cisplatin?

Cisplatin can be replaced by carboplatin according to the integrated management clinical approach in other cancers, but for this setting, we still do not have enough data to recommend carboplatin-based regimens for NAC in BC [5,6]. A consensus definition of pts with metastatic urothelial carcinoma that unfit for cisplatin-based chemotherapy was released in 2011 among BC experts. Pts “unfit” for cisplatin were defined with at least one of the following criteria: performance status > 1; glomerular filtration rate < 60 mL/min; grade > 2 audiometric loss; peripheral neuropathy and New York Heart Association (NYHA) class III heart failure [7]. It appears clear that the probability of ineligibility to cisplatin increases with age because, by the Cockroft–Gault equation for measurement of renal function, more than 40% of pts with an age over 70 years are ineligible, considering that the average age of BC is around 70 years. Many randomized trials have shown that carboplatin-based therapy is inferior compared with cisplatin in terms of OS and pCR; that is why some authors proposed a more tolerated reduced dose of cisplatin 50 mg/m^2^: a sequential ifosfamide, doxorubicin, and gemcitabine followed by reduced-dose cisplatin, gemcitabine, and ifosfamide resulted in similar oncological outcomes, with a pathological downstaging to pT1N0 disease or lower occurring in 50% of pts who underwent RC [44,45]. Despite these data, nowadays in cisplatin-ineligible pts, the standard of care remains to be upfront RC, unfortunately with a higher risk of systemic relapse [5,6]. We speculate that other biological agents such as neoadjuvant immune checkpoint inhibitors (ICIs) could later be the solution for this subset of pts.

### 3.4. Which Is the Right Timing for Surgery?

It is important to not delay surgery, especially in pts that will not respond, so treatment delays could lead to an increased difficulty of the surgical procedure due to perioperative morbidity. In fact, actually one of the main reasons for low uptake of NAC is the risk in increasing the timing between diagnosis of MIBC and surgical treatment because such a delay has been shown to negatively impact oncological outcomes. This statement is based on a meta-analysis pooling 12 retrospective and 1 prospective trials that failed to show a linear relationship between delay and prognosis, but the majority confirmed that a longer delay was associated with worse outcomes, with a window of opportunity of less than 12 weeks from the diagnosis [46]. Nevertheless, a Dutch trial did not show any difference in clinical outcomes after adjusting for confounding variables between delayed RC more than 3 months from the diagnosis and traditional RC, so optimal timing of cystectomy is still far to be defined. It is worthy to underline that, different from primary MIBC, a secondary MIBC is not likely to respond to NAC and should always be considered for upfront total cystectomy [47].

### 3.5. Are There More Complications for Surgery after Neoadjuvant CT?

Although the administration of neoadjuvant CT increased MIBC overall survival, only 15% of pts who underwent RC received neoadjuvant CT [48,49,50,51]. Among the reasons that could explain this phenomenon, concerns regarding detrimental perioperative complications could have limited this approach in eligible pts. As reported by Gandaglia et al., the use of neoadjuvant chemotherapy is, however, not associated with higher perioperative morbidity and mortality. Indeed, no significant differences were observed in rates of complications, readmissions, and mortality among pts who received neoadjuvant CT and RC compared with RC alone [52]. A similar result was obtained by Tyson et al., who reported no difference in perioperative complication at surgery for pts receiving neoadjuvant CT, although a higher rate of peripheral nerve deficits (mostly related to the use of cisplatin-based chemotherapy) was found [53]. Moreover, neoadjuvant CT was not associated with increased operative time or wound infection rate and could aid the surgeon in converting unresectable disease to operable tumor burden [54]. Considering the new adjuvant CTs such as Atezolizumab and Pembrolizumab, the results were similar, confirming the tolerability and the absence of impact in the surgical setting, as reported in the safety results from ABACUS trial [55] and PURE-01 trial [56]. In particular, the most common complications were limited to minor grade complications as fever (35%) and ileus (21%), while major grade complications (Clavien Dindo > 3a) were consistent with other pts who received RC alone (20–30%) [56].

### 3.6. How to Manage Variant BC?

Another unmet need about MIBC is how to manage variant BC. As shown in EAU-ESMO consensus statements, BC with small-cell neuroendocrine variant should be treated with neoadjuvant chemotherapy followed by consolidating local therapy; muscle-invasive pure-squamous cell carcinoma and pure-adenocarcinoma of the bladder should be treated with primary RC and lymphadenectomy [5]. In a subgroup analysis from the randomized controlled trial, SWOG-8710 pts with mixed histology—squamous or glandular—were seen to have a survival benefit from NAC compared with primary RC (HR 0.46; 95% CI 0.25–0.87; *p* = 0.02) [57], consistent with a previous report that showed similar oncological outcomes for pts with pure urothelial BC and variant histology treated with NAC, with a higher rate of pathological downstaging too [58]. For micropapillary histology, authors reported pathological complete response rate after NAC ranging from 11% to 55% without significantly affecting relapse-free survival (RFS) (HR 1.23; 95% CI 0.52–2.93; *p* = 0.6) or OS (HR 1.35; 95% CI 0.98–1.86; *p* = 0.1) [59]. Plasmocytoid variant is very rare and aggressive, but data are immature for considering NAC [60]. Moreover, urachal carcinoma has been shown to have no benefit with cisplatin-based neoadjuvant chemotherapy [61].

## 4. Neoadjuvant Immunotherapy

Tumor cells evade immunosurveillance and progress towards a malignant behavior through different mechanisms [62]. Immune checkpoint inhibitors (ICIs) reinvigorate antitumor immune responses by interrupting coinhibitory signaling pathways and promoting immune-mediated tumor cells elimination. Monoclonal antibodies blocking the immune checkpoint through PD-1, PD-L1, or CTLA-4 have demonstrated impressive clinical activities against several tumors [63,64].

PD-1 on T cells is activated by its ligand PD-L1 expressed by diverse cell types. PD-L1/PD-1 binding leads to T cell response inhibition. Under physiological conditions, PD-1 expression on T cells is induced by TCR signaling and its expression decreases to basal levels upon antigen clearance. Persistent antigen stimulation in the tumor microenvironment can lead to constitutively high PD-1 expression [65]. CTLA-4 expression and function are linked to T cell activation, reducing TCR signaling by competing with the costimulatory molecule CD28 for the B7 ligands (B7-1/CD80 and B7-2/CD86) [66,67].

ICIs have been used for metastatic BC treatment as a second-line therapeutic approach after platinum-therapy failure [64] or as first-line strategy for cisplatin-ineligible pts [68]. In addition, the anti-PDL1 avelumab has been approved in maintenance setting after first-line chemotherapy [69].

More recently, ICIs have shown promising results in MIBC preoperative setting, in terms of both pathological response and safety profile. Several phase II trials highlighted the potential role of immunotherapy as a valuable strategy for neoadjuvant therapy, especially in cisplatin-unfit pts [70].

PURE-01 [56] was the first open label, single-arm, phase II study evaluating the neoadjuvant pembrolizumab efficacy in MIBC pts, along with biomarker analysis. All enrolled pts underwent RC after treatment, with 42% showing pathological complete response (pT0). This response was significantly enriched in pts with PD-L1 combined positive score (CPS) ≥ 10%. Pembrolizumab was well tolerated with few immune-related adverse events (AEs). Interestingly, pT0 was associated with DNA damage response (DDR) gene alterations and high tumor mutational burden (TMB), suggesting a predictive role of these two biomarkers.

Consistently with these results, anti-PDL1 Atezolizumab showed interesting outcomes as a neoadjuvant treatment for MIBC in the ABACUS trial [55], with a pathological complete response rate of 29% (95% CI: 18–42%) and good safety. In fact, grade 3–5 AEs occurred only in 11% (10/95) of pts treated with atezolizumab [71].

Anti-PD1 Nivolumab and anti-CTLA4 Ipilimumab combination was also assessed in preoperative setting in the NABUCCO trial [72]. A complete response was observed in 46% of pts, irrespective of pre-existing CD8^+^ T cell activity. Moreover, DUTRENEO [73] and MDACC [74] trials aimed to assess the Durvalumab plus Tremelimumab activity before RC. In the former trial, pts were selected according to tumor immune score (TIS) and randomized to receive standard CT or combination with Durvalumab + Tremelimumab. The immunotherapy group showed a complete pathological response rate of 34.8%, with a safe profile. However, TIS did not seem to correlate with the response in this group.

The phase II BLASST trial [75] tested the Nivolumab and GC combination as neoadjuvant treatment for MIBC. A complete pathological response was observed in 65.8% of pts, including those with N1 disease, and grade 3–4 AEs in 20% of study population, mostly from GC.

Finally, the phase 3 trial ENERGIZE [76] evaluated the efficacy and safety of GC alone or with Nivolumab or Nivolumab + Linrodostat (an IDO1 inhibitor) in the neoadjuvant setting. The blockade of these two pathways could be a solid alternative in cisplatin-ineligible pts. Table 2 summarizes the main trials evaluating neoadjuvant immunotherapy for BC.

## 5. Discussion and Conclusions

Neoadjuvant platinum-based chemotherapy represents the standard treatment for non-metastatic MIBC [77]. However, cisplatin-ineligible pts could not benefit from a systemic therapy before surgery, with a high risk of relapse after RC. In this scenario, many trials evaluating the role of ICIs have shown promising results. Despite the good outcomes considering pCR and OS/PFS, pts that could benefit from this treatment have not been identified yet. As a consequence, molecular biomarkers for patient’s selection are needed. As described before, many trials investigated the role of PD-L1 or TMB with promising evidence.

DDR alterations have been correlated to immunotherapy response in several cancer types. Considering that BC is highly reliant on DDR alterations and activation of genes involved in intrinsic DNA damage and replication stress (such as p53 and Rb), we foresee that BC could be an ideal setting to test combinations/sequence of chemotherapy or DDR targeting agents or radiotherapy with immunotherapy in order to increase antitumor immune response. However, further investigations are needed to verify the efficacy of this approach.

The rational for these combinations is based on the ability of cancer cells with high intrinsic or treatment-induced DNA damage to activate an innate immune response via STING (Stimulator of interferon response), as demonstrated in other cancer types [78,79].

In this context, we speculate that circulating tumor DNA (ctDNA) analysis could be useful to identify valid biomarkers, as emerged from the Imvigor010 trial [80,81]. However, data from the ctDNA analysis showed interesting results [82]. ctDNA levels have been used as valid predictors of complete tumor response in multiple cancer types [83]. Pts positive for ctDNA show higher OS and PFS in the immunotherapy arm than the observation arm (DFS HR, 0.58 (95% CI: 0.43–0.79) *p* = 0.0024; OS HR, 0.59 (95% CI: 0.41–0.86)) [82]. In conclusion, these results could be useful to modify clinical practice in the neoadjuvant setting, although further clinical trials and evaluation of molecular biomarkers are needed for better pts’ selection.

## Figures and Tables

**Table 1:** Summary of trials using chemotherapy in neoadjuvant setting.

Trial Name	Phase	NAC	Regimen	Elegible Pts	Sample Size	pCR	DFS	OS
Wallace et al., 1991 + Raghavan et al., 1991	Randomized clinical trial	Cisplatin→Cystectomy vs. RT	//	Elegible for CDDP	255	//	//	No difference between two arms
Martinez-Pinerio et al., 1995	Randomized clinical trial	Cisplatin→Cystectomy vs. Cystectomy	//	Elegible for CDDP	121	//	//	No difference between two arms
EORTC/MRC BA06, 1999	Randomized clinical trial	CMV→Cystectomy/RT vs. Cystectomy/RT	//	Elegible for CDDP	976	//	//	5 y OS 49% in CMV group vs. 43% in Cystectomy group (*p*-value 0.048)
Bassi P. et al. (GUONE), 1999	Randomized clinical trial	M-VAC→Cystectomy vs. Cystectomy	4 Cycles q 28 M-VAC	Elegible for CDDP	206	//	//	No difference between two arms
H. Barton Grossman, et al. (SWOG-8710), 2003	Randomized controlled trial	M-VAC→Cystectomy vs. Cystectomy	3 Cycles q 28 M-VAC	Elegible for CDDP	307	38%	//	5 y OS 57% vs. 43% (*p*-value 0.06): NAC’s favour trend, but not statistically significant
GISTV (Italian Bladder Cancer Group), 1996	Randomized clinical trial	M-VEC→Cystectomy vs. Cystectomy	3 cycles MVEC	Elegible for CDDP	171	24%	//	No difference between two arms
H. von der Maase et al., 2000	Randomized clinical trial	CDDP + Gem→cystectomy vs. MVAC→Cystectomy	Max 6 Cycles CDDP + Gem q28 or MVAC q28	Elegible for CDDP	405	12.5% in CDDP + Gem group vs. 11.9% in MVAC group	mDFS 7.4 m in both groups	mOS 13.8 m vs. 14.8 m (*p*-value 0.75). CDDP + Gem was superior in risk–benefit ratio with no difference in OS
Sherif A. (NORDIC I–II), 2004	Randomized clinical trial	ADM + CDDP + RT→cystectomy vs. RT + Cistectomy MTX + CDDP→cystectomy vs. Cystectomy	3 cycles q 28	Elegible for CDDP	620	//	//	5 y OS 56% in NAC group vs. 48% in CYstectomy group (*p*-value 0.045)
Osman et al., 2014	Randomized clinical trial	CDDP + Gem→Cystectomy vs. Cystectomy	3 cycles q21	Elegible for CDDP	60	35%	3 y DFS 57% in NAC group vs. 43% in Cystectomy group	3 y OS 60% in NAC group vs. 50% in Cystectomy group
Kitamura et al., 2014 JCOG0209	Randomized clinical trial	M-VAC→Cystectomy vs. Cystectomy	2 cycles M-VAC q 28	Elegible for CDDP	130	34%	99 m in NAC group vs. 78 m in cystectomy group	5 y OS 72.3% in NAC group vs. 62.4% in Cystectomy group
GETUG/AFU V05 VESPER (2021)	Randomized clinical trial	ddMVAC→Cystectomy vs. CDDP + Gem→Cystectomy	6 cycles ddMVAC q14 vs. 4 cycles CDDP + Gem q21	Elegible for CDDP	437 in neoadjuvant setting	42% in ddMVAC group vs. 36% in CDDP + Gem group (*p*-value 0.021)	3 y PFS 66% dd MVAC vs. 56% CDDP + Gem (*p*-value 0.025).	OS data are not yet ready, but are expected to confirm these results.

Abbreviations: NAC—neoadjuvant chemotherapy; pts—patients; pCR—pathological complete response; DFS—disease-free survival; OS—overall survival; RT—radiotherapy; CDDP—cisplatin; M-VAC—methotrexate, vinblastine sulfate, adriamycin, and cisplatin; CMV—cisplatin methotrexate, vinblastine sulfate; MVEC—methotrexate, vinblastine, epirubicin, and cisplatin; ADM—Adriamycin; MTX—methotrexate; ddMAVC—dose-dense M-VAC.

**Table 2:** Summary of trials using immunotherapy in neoadjuvant setting.

Trial Name	Phase	Drug	Regimen	Elegible Pts	Sample Size	pCR Rate/pCR Rate PD-L1+	DFS/DFS PD-L1+	OS
PURE-01	2	Pembrolizumab	3 cycles	Elegible and ineligible for CDDP	143 pts	55%/54.3%	1 y DFS 84%	1 y OS 91%
ABACUS	2	Atezolizumab	2 cycles	Ineligible for CDDP	88 pts	27%/37%	79%/75–95%	NA
NABUCCO	2	Nivolumab + Ipilimumab	2 cycles	Ineligible or refused CDDP	24 pts	11%/73%	NA	NA
DUTRENEO	2	Durvalumab + Tremelimumab vs. CT (Gem + CDDP or MVACdd)	3 cycles	Elegible for CDDP	61 pts (enrolling)	36,4% vs. 34.8% /PDL1 high 57.1% vs. 60%/PDL1 low 14.3% vs. 60%	NA	NA
MDACC	1	Durvalumab + Tremelimumab	Du + Tre × 2 clycles OR Du + Tre × 1 cycles →Du × 1 cycles	CDDP ineligible	28 pts (enrolling)	37.5%	NA	NA
BLASST	2	Nivolumab + CDPP + Gem	4 cycles	Elegible for CDDP	41 pts	34%/NA	NA	NA
ENERGIZE	3	CDDP + Gem vs. CDDP + Gem + Nivolumab + Placebo vs. CDDP + Gem + Nivolumab + Linrodostat mesylate	4 clycles OR 4 clycles→9 cycles (adjuvant)	Elegible for CDDP	1200 pts (enrolling)	NA	NA	NA

Abbreviations: pCR—pathological complete response; DFS—disease free survival; OS—overall survival; pts—patients; CDDP—cisplatin; ddMAVC—dose-dense M-VAC (methotrexate, vinblastine sulfate, adriamycin, and cisplatin; NA—not available; Du—Durvalumab; Tre—Tremelimumab.

## Data Availability

Not applicable.
